# Macular Pigment Optical Density Measured by Heterochromatic Modulation Photometry

**DOI:** 10.1371/journal.pone.0110521

**Published:** 2014-10-29

**Authors:** Cord Huchzermeyer, Juliane Schlomberg, Ulrich Welge-Lüssen, Tos T. J. M. Berendschot, Joel Pokorny, Jan Kremers

**Affiliations:** 1 University Eye Hospital Erlangen, Friedrich-Alexander-University Erlangen-Nürnberg, Erlangen, Germany; 2 Department of Ophthalmology, Charité, University Medicine Berlin, Berlin, Germany; 3 University Eye Clinic Maastricht, Maastricht, The Netherlands; 4 Department of Ophthalmology and Visual Sciences, University of Chicago, Chicago, Illinois, United States of America; Univ Rochester Medical Ctr, United States of America

## Abstract

**Purpose:**

To psychophysically determine macular pigment optical density (MPOD) employing the heterochromatic modulation photometry (HMP) paradigm by estimating 460 nm absorption at central and peripheral retinal locations.

**Methods:**

For the HMP measurements, two lights (B: 460 nm and R: 660 nm) were presented in a test field and were modulated in counterphase at medium or high frequencies. The contrasts of the two lights were varied in tandem to determine flicker detection thresholds. Detection thresholds were measured for different R:B modulation ratios. The modulation ratio with minimal sensitivity (maximal threshold) is the point of equiluminance. Measurements were performed in 25 normal subjects (11 male, 14 female; age: 30±11 years, mean ± sd) using an eight channel LED stimulator with Maxwellian view optics. The results were compared with those from two published techniques – one based on heterochromatic flicker photometry (Macular Densitometer) and the other on fundus reflectometry (MPR).

**Results:**

We were able to estimate MPOD with HMP using a modified theoretical model that was fitted to the HMP data. The resultant MPOD_HMP_ values correlated significantly with the MPOD_MPR_ values and with the MPOD_HFP_ values obtained at 0.25° and 0.5° retinal eccentricity.

**Conclusions:**

HMP is a flicker-based method with measurements taken at a constant mean chromaticity and luminance. The data can be well fit by a model that allows all data points to contribute to the photometric equality estimate. Therefore, we think that HMP may be a useful method for MPOD measurements, in basic and clinical vision experiments.

## Introduction

The macula lutea (“yellow spot”), located in the central retina, derives its name from its yellowish appearance due to blue light absorption by the macular pigment (MP). The latter consists of the carotinoids lutein and zeaxanthin[1]–[3] and absorbs short-wavelength light with a peak optical density at 460 nm [4]. The biological function is hypothesized to be a protection of the retina against phototoxicity by shielding against short-wavelength light [5], and by anti-oxidative effects [6], [7]. In some studies, it is suggested that MP enhances visual performance [8], [9]. Macular pigment optical density (MPOD) varies substantially between observers [10]. Assessing the individual MPOD may therefore have clinical relevance for estimating the individual protective effects. Large intra-individual variability of MPOD is also relevant in psychophysical experiments because of variation in the available short-wavelength light at the receptoral level.

MPOD can be measured with psychophysical or physical methods. Psychophysical methods compare the individual sensitivity to short-wavelength light in the macula (where MP is present in high concentration) and at a peripheral retinal location (where MPOD is low or absent) [11]. This sensitivity for short-wavelength light is usually measured relative to the sensitivity to a longer wavelength light (not absorbed by the MP), by matching the two lights in luminance. The common method for making such photometric matches is heterochromatic flicker photometry (HFP)[12]–[14]. In HFP, the two lights are alternated in counterphase at temporal frequencies typically between 10 and 20 Hz [13] at 100% contrast. The observer adjusts the time-averaged luminance of one of the lights until the perception of flicker is absent or minimized. The luminance level of the test light that gives the perception of minimum flicker for the observer is taken as the luminance level that is equal to the standard light luminance.

Although it has been shown that HFP can be successfully used to measure MPOD[12]–[14], it has some drawbacks. First, the task of minimizing flicker by adjusting the luminance is difficult to understand for inexperienced observers [15], [16]. With HFP the observer must find a point of minimum flicker by adjusting the luminance of one light that is alternating with another light. Adjusting the luminance of the light either too high or too low increases the perception of flicker and the observer sometimes may become confused. Second, the characteristics of the flicker-null-zone are highly dependent on the flicker frequency: on one hand, large flicker-null-zones are observed when the frequency is too high and this can lead to inaccurate matches. On the other hand, a flicker null may be absent if the frequency is too low. Third, the precision of the results cannot be monitored easily (and can be only estimated by the variability of a series of measurements). Finally, in the original HFP paradigm the average luminance of only one light is changed: thus the mean luminance and chromaticity of the test field are continuously changed, which can influence the results of the measurements [17]. Some of these drawbacks can be overcome by modifications to the HFP paradigm. For example, the change in mean luminance can be avoided by changing the luminance of both lights (inverse-yoking)[18]–[20]. However, the change in mean chromaticity is inherent to the technique.

**Figure 1:**
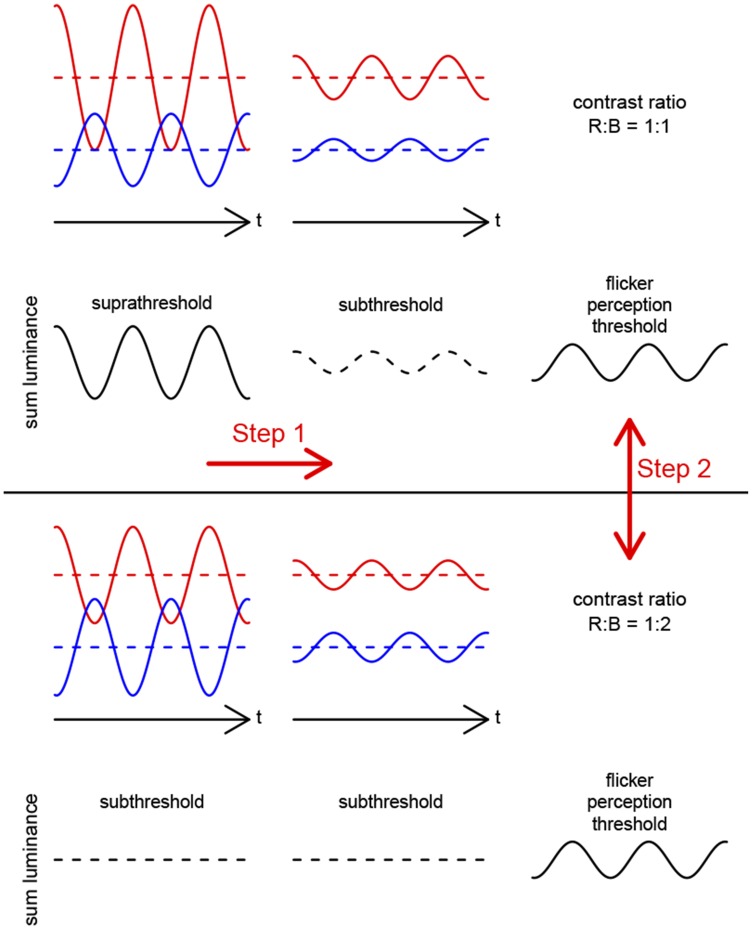
Illustration of the basic principle of HMP. Luminance is plotted as a function of time as the 660 nm (red) and the 450 nm (blue) test lights (red/blue curves) are alternated in counterphase. The resulting sum luminance is shown below (black curves). In these examples, the luminance ratio red:blue is always 2∶1. First, the modulation depths of both lights are reduced in tandem (so that the modulation ratio is constant) and a threshold is determined. Then, this procedure is repeated for different modulation ratios and (only two exemplary modulation ratios, 1∶1 and 1∶2, are shown here). Note that in the lower example, the modulation ratio is 1∶2, and the theoretical sum luminance is always zero. In practice, a threshold can be determined even in this case, and this may be mediated by mechanisms other than the luminance pathway.

Heterochromatic *modulation* photometry (HMP) is a modification of Heterochromatic *Flicker* Photometry that was developed by Pokorny and coworkers [15], [17], [21] to overcome some of the limitations of HFP. Like in HFP, a test and a reference light are modulated in counterphase at medium or high frequencies. A series of fixed standard/test modulation ratios are presented, and at each ratio the modulation depth of the pair is reduced in tandem until the observer reports that flicker disappears. The expectation is a distinct minimum modulation sensitivity at the standard/test ratio representing the luminance match. At other modulation ratios, flicker sensitivity should vary with the luminance difference between standard and test. The data can be fit by theoretical templates to describe modulation sensitivity as a function of standard/test ratio (see Figure 1). A major advantage of this paradigm is the fact that the mean luminances of the two lights (and thus also the mean overall luminance and chromaticity) remain constant throughout the experiment. Further, the task can be easily performed by naïve observers because the measurement involves the determination of a flicker detection threshold rather than a flicker null. In addition, the relationship between modulation ratio and sensitivity can be described by a theoretical function, with which the minimum point can be identified using all data points [15], [17].

It is the purpose of the present study to determine MPOD by performing HMP measurements at central and peripheral retinal locations using an LED stimulator with Maxwellian view optics [22] that is also used for psychophysical experiments. The initial purpose was to perform psychophysical MPOD measurements with this apparatus to correct for inter-individual variation in MPOD in future experiments using the same apparatus. The results of the measurements were compared with MPOD data obtained with the conventional psychophysical HFP method [23] and with fundus reflectometry [24]. Using an improved version of the original mathematical model for the relationship between sensitivity and contrast ratio [15], [17] allowed precise estimation of MPOD.

The data show that the HMP method can be an easy to perform and relatively quick method to obtain macular pigment density that can be used to correct for individual differences in preretinal absorption when using the same equipment for studying retinal function.

## Methods

We used heterochromatic modulation photometry to measure macular pigment optical density and compared the results with those of two established methods: heterochromatic flicker photometry – a psychophysical method – and macular pigment reflectometry – a technique based on measuring light reflected from the retina. An augmented version of the model proposed by Pokorny, Smith and Lutze [15], [17] was fit to the HMP data.

### Heterochromatic Modulation Photometry

#### Apparatus

For this experiment, we used an eight channel LED stimulator with Maxwellian view optical system developed by Pokorny et al. [22] for selective stimulation of photoreceptor types (including rods) [25], [26]. Technical details, including a photograph and the optical layout, can be found in the original report [22]. Briefly, two channels, each containing four LED-interference filter combinations, produced 8–10 nm bandwidth primaries with emission spectra maxima at 660 [red], 558 [green], 516 [cyan] and 460 nm [blue]). One channel produced a central 2° diameter stimulus; the second an annulus with a 2° inner and 13° outer diameter. The 2° diameter for the central stimulus was chosen for the original purpose of the LED stimulator (i.e. experiments controlling the stimulation of the four receptor types individually). The stimulus field was viewed through a 3 mm diameter artificial pupil. The luminance of the LEDs were controlled by a Desktop PC with a Xonar D2-PM sound card (ASUSTek Computer Inc., Taipei, Taiwan). The amplitude-modulated signal of the sound card (20 kHz carrier wave) was converted by a voltage-to-frequency converter (Texas Instruments VFC320CP) into a frequency-modulated signal (up to 250 k Hz) [27]. Fourth-order polynomials were used to describe the relationship between output of the soundcard and light output of the LEDs. During calibration, light output was measured for 13 different output levels of the soundcard (1%, 5%, 10%, 20%, …, 80%, 90%, 95% and 99% of maximal output). Radiometric calibrations were performed with a CAS 1401 spectroradiometer (Instrument Systems). Relative photometric outputs were obtained using a 268R illuminance sensor (UDT Instruments, Baltimore, Maryland) whose current was measured with an International Light IL1700 radiometer. Retinal illuminance was calculated using the method described by Nygaard and Frumkes after converting the radiometric measurements to photometric values [28].

#### Software

The LEDs were controlled using custom-made software developed using C# that is driving the output of the sound card allowing adjustment of the luminance and modulation of each LED as described previously [22]. The software was designed to determine flicker detection thresholds in the center or surround field using a forced-choice (yes/no)-paradigm with randomly interleaved staircases, one starting at maximal modulation and the other starting at zero modulation [29], [30]. Observers indicated verbally whether or not they perceived a flicker in the test field. Initial step size of modulation was set to one fifth of the maximal modulation. The contrast was decreased when flicker was perceived and it was increased when no flicker was perceived. At each change in perception (from “flicker” to “no flicker” and *vice versa*) the contrast step was halved. Termination criterion was a contrast step of less than 1/7th of the actual contrast. When the observer indicated three times no flicker perception at maximal contrast, it was assumed that threshold could not be obtained and the measurement was interrupted. The initial conditions were documented in preset files that were created on the fly using scripts in the R language (see subsection on statistical analysis) and were read at the beginning of a measurement. The advantage of this approach was that the stimulus conditions could be adapted quickly to individual circumstances (i.e. depending on estimates of the observers' macular pigment density or overall sensitivity). Data analysis and presentation were also performed using custom-made R scripts.

#### Conditions and protocol

In our experiments, the 2° center field was used as the test field: the blue primary (460 nm; half-height bandwidth: 8 nm) and the red primary (660 nm; half-height bandwidth: 10 nm) were modulated in counterphase. Each LED produced a time-averaged retinal illuminance of 21 Td, yielding an average retinal illuminance of 42 Td.

For the central measurement, the observer was instructed to fixate a point in the center of the test field. For the peripheral measurement, there was a fixation point at a 6° eccentricity in the nasal visual field. The fixation points were created by entering an otherwise transparent slide into the optical pathway close to the retinal plane of the stimulator. To suppress influence of stray light and to saturate rods, there was a steady bright white appearing surround field (13° visual angle, retinal illuminance: 587 Td, 2°CIE coordinates x = 0.34, y = 0.31). The experiments were performed after the observers adapted to dim room light for at least 2 minutes.

**Figure 2:**
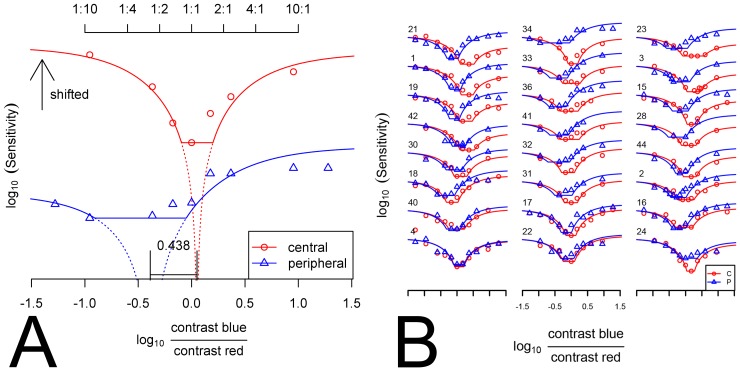
Measurement of macular pigment optical density using HMP. A) Determination of the macular pigment optical density (MPOD) from the photometric matches. The modulation sensitivity (1/threshold) curves for central and peripheral fixation are plotted against modulation ratio on a log-log-scale. The curve for central fixation is shifted upward for better visualization (black arrow). The minimum of the modulation sensitivity curves is determined by fitting a theoretical function to the data. Because the CIE luminances [38] of both lights are identical, the position of the flicker minimum on the ordinate is zero for an observer with a luminous sensitivity identical to the CIE standard observer's. A shift of the minimum of the modulation sensitivity curve to the left indicates a higher sensitivity for the 460 nm light, a shift to the right indicates a lower sensitivity. The MPOD (expressed in optical density units at 460 nm) is calculated by subtracting the minimum flicker point for peripheral fixation from that obtained for central fixation. B) Modulation ratio-sensitivity curves together with the fitted models for all 24 observers. The curves measured under central (C) and peripheral (P) absorption were shifted vertically, so that the position on the Y-axis at x = −1.5 for both curves of each observer is equal. For facility of inspection, the curves for the individual observers were ordered in a way that MPOD increases from left to right and from bottom to top.

At each retinal location, flicker detection thresholds were measured for different modulation ratios of the red and blue LEDs. The LEDs were modulated sinusoidally in counterphase. Initially, the frequency was set to 18 Hz for central measurements and to 16 Hz for peripheral measurements. The first measurements, performed with modulation of only one of the two primaries, corresponded to logarithmic modulation ratios of ± infinity and marked the asymptotes of the sensitivity function (see Figure 2). If the observer's temporal contrast sensitivity was low (defined as the two thresholds were both larger than 20% contrast), the test frequency was reduced and the measurements repeated until a satisfactory sensitivity was reached. The frequencies that were determined this way were then used during the rest of the experiment. Subsequently, threshold measurements were performed for 460 nm/660 nm contrast ratios of 1∶9, 3∶7, 4∶6, 5∶5, 6∶4, 7∶3 and 1∶9 (corresponding to log modulation ratios of −0.95, −0.37, −0.18, 0, 0.18, 0.37, and 0.95). If a minimum was close to a log modulation ratio of either ±1, measurements were performed at more extreme modulation ratios. A condition was repeated if the two staircase thresholds differed markedly from each other. The sensitivity at each contrast ratio was calculated by dividing the maximal LED contrasts by those at threshold. Finally, the minimum of the modulation sensitivity vs. log contrast ratio function was identified by fitting a theoretical function with a least-squares method using the nls routine (nls = Nonlinear Least-Squares) of the R statistics package with the Newton-Gauss algorithm. The mathematical function was modified from the one used by Pokorny et al. [15], [17] (see Appendix S1 for its derivation). As seen in Figure 2, the MPOD is calculated from the differences between the minima obtained from central and eccentric measurements. Visual control of the fitted curve showed that the fit was well constrained by the data.

### Heterochromatic Flicker Photometry

MPOD estimates were also obtained using HFP with the Ocular Densitometer (Macular Metrics, Rehoboth, USA) using previously described methods [10], [23]. The observer adjusted the mean luminance of a blue (460 nm) test light that was alternated in counterphase with a green (550 nm) reference light, to minimize flicker perception. The mean luminance of the reference light was changed inversely relative to the test light but by the same magnitude, so that time-averaged luminance (but not time-averaged chromaticity) remained constant. Flicker frequency was initially chosen according to recommendations provided by the manufacturer that considered the observer’s age and was subsequently adjusted when the variability of sequential readings was high, or when the observer reported either an absent or a wide null-flicker zone. Three consecutive measurements were obtained at each eccentricity (0.25°, 0.5°, 1.0°, 1.75°) and for the peripheral reference point at 7° retinal eccentricity. MPOD was estimated from the difference of the central and peripheral values.

### Macular Pigment Reflectometry

MPOD estimates were also obtained using the Macular Pigment Reflectometer (Maastricht Instruments bv, the Netherlands) according to methods described by Van der Kraats, Van Norren, Berendschot et al. [24], [31], [32]. Briefly, the spectrum of the reflection of a white spot with 1° diameter (400 to 880 nm with known spectrum) centered on the fovea was measured. The resultant spectral reflectances were analyzed using a theoretical model based on assumptions about reflections and absorptions at different layers in the human eye (see appendix in Van de Kraats [32]). Parameters for lens optical density were estimated from the observer’s age. At least five measurements were obtained with natural pupil. Measurements with large error bars (indicating large variation in the reflectance curve) or with disruptive corneal reflections in the short-wavelength spectrum were discarded solely by the appearance of the reflectance curves immediately after they were obtained and before the extraction of MPOD information. The average number of discarded measurements was 5.6 (standard deviation 3.7).

### Observers

Twenty-five volunteers (11 male, 14 female; age: 30±11 years, mean ± sd; range: 20–58 years; all Caucasian) were recruited from the staff of the University Eye Hospital Erlangen and the medical students of the University Erlangen-Nürnberg. The study was approved by the ethics committee of the medical faculty of the university (Ethik-Kommission der Friedrich-Alexander-Universität Erlangen-Nürnberg). All observers gave informed written consent, and all experiments adhered to the tenets of the Declaration of Helsinki.

All observers underwent clinical examinations of best-corrected visual acuity, slit-lamp examination, funduscopy and optical coherence tomography of the central retina. The psychophysical examinations were performed before the MPR measurements.

### Statistical analysis

Statistical analyses were performed using the free open-source software R (R Development Core Team (2012): R: A language and environment for statistical computing. R Foundation for Statistical Computing, Vienna, Austria, http://www.R-project.org/).

Primary outcome measures were feasibility of measurements and the correlation of measured MPOD with measurements by HFP at any of the four locations (Bonferroni corrected for multiple testing) and by MPR. The distributions of all variables were tested for normality using the Shapiro-test. Correlation was described by Pearson's R.

Secondary analyses were performed to investigate the agreement between the established methods, and to correlate MPOD_HMP_ and MPOD_HFP_ with lutein and zeaxanthin measurements by MPR. Statistical significance was considered to be reached for p-values of ≤0.05.

## Results

MPOD_HMP_ could be measured in 24 of the 25 observers. The data for one observer were too variable to constrain the theoretical function fit, and the data were excluded from analysis. In the MPOD_MPR_ measurements data from another observer exhibited high variability (9 readings between 0.32 and 0.91, standard deviation 0.24), and model parameters were not well constrained. In a third observer, HFP measurements were not performed. Thus, comparisons between HMP and MPR, HMP and HFP, and MPR and HFP are each based on 24 observers.

The raw data from the HMP measurements are provided in Table S1. This table contains the results from all threshold measurements. Table S2 contains the HFP and MPR data and some of the clinical data. The variable names used in both tables are explained in Table S3.

### Measuring MPOD with HMP

#### Threshold Measurements

For the central retina, frequencies between 12 and 16 Hz yielded precise data (16 Hz: n = 14; 15 Hz: n = 1; 14 Hz: n = 6; and 12 Hz: n = 3). Time to complete the foveally fixated measurements was 10.8±5.9 min. Observers then performed the test while looking at the peripheral fixation point. Some observers had initial difficulties in maintaining stable fixation. In addition, some observers were sometimes troubled by Troxler’s fading. Often, the administrator was able to identify inconsistent fixation on a trial by noting large differences between the two interleaved staircases thresholds. Although some trials were repeated, the average duration of the peripheral measurements was shorter, 8.7±2.5 min. Data at the predefined 460 nm/660 nm contrast ratios were obtained at frequencies between 6 and 16 Hz (16 Hz, 14 Hz: n = 1; 12 Hz: n = 11; 10 Hz: n = 7; 8 Hz, 6 Hz: n = 1).

#### Fitting of asymmetric theoretical curves using a revised model

**Figure 3:**
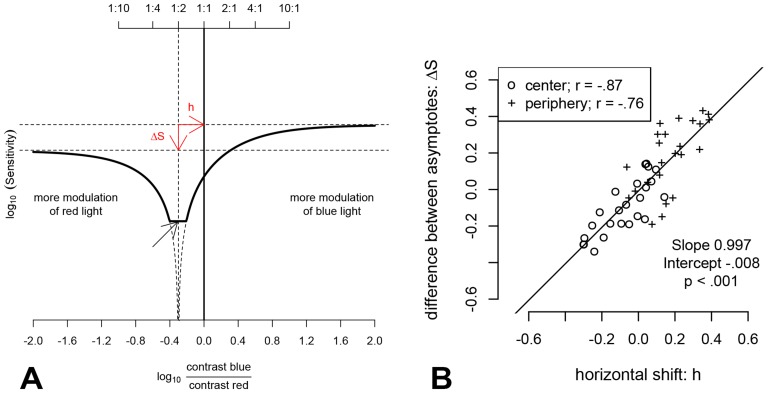
Modeling the modulation sensitivity functions. A) A revision of the Pokorny, Smith and Lutze model [15], [17] was fit to the modulation sensitivity threshold data (see Figure 2b for original data and model fits). The derivation of the model is described in detail in Appendix S1. In contrast to the original HMP function, the shape of the new function varies with changes in the horizontal position of the equiluminant point. A prediction from the model is that the difference between the heights of the asymptotes of the modulation sensitivity function (ΔS) is equal to the horizontal shift of the minimum flicker point (h). B) The difference in the asymptotes for this theoretical relationship is evident in our data.

In Figure 2A, two data sets of one observer are displayed together with fits of the model to the data. The lower data points (with peripheral fixation) are clearly asymmetrical around the minimum. Such asymmetries were often observed in our data (Figure 2B), particularly in cases where the minimal sensitivities occurred at log(contrast ratios) that deviated substantially from zero. This asymmetry was not accounted for by the original model described by Pokorny and coworkers [15]. Similar asymmetric data were, however, obtained in a second paper on HMP by Pokorny et al. [17], where the two lights had been set to different CIE luminances to achieve different chromaticities of the test field. The asymmetries in the present data are likely caused by the fact that the absence of the macular pigment in peripheral measurements resulted in a higher relative luminance of the blue light at the photoreceptor level. This led to a shift of the equiluminant point to the left (as we had expected), and also to higher sensitivities at the right side of the curve, where flicker was dominated by the blue light. Therefore, the extent of asymmetry was directly related to the horizontal distance between the equiluminant point and the zero-point. We modified the original model by accounting for the absorption changes (a derivation of the modified model can be found in Appendix S1). This modified model could account for the observed shape of the function coupled to the position of the minimum along the log(contrast ratio) axis (see also Figure 3). Figure 2B shows all data points from the central (red circles) and peripheral measurements (blue triangles) of all subjects together with the fitted curves (red/blue lines). It can be seen that the modified model provides a satisfactory description of all data sets since it captures several features in the data: the change in sensitivity as a function of the log(contrast ratio), the position of minimal sensitivity and the asymmetry in asymptotes. The data sets of the subjects with high MPOD can be found toward the top of the diagram and curves were shifted vertically, so that the position on the Y-axis at x = −1.5 for both curves of each observer was equal. Therefore, by looking at the right hand side of the individual curves, one can appreciate that the revised model accounts for the asymmetries that we encountered.

The revised model may offer another potentially interesting extension. On the basis of the revised model, it can be deduced that the difference in height between the asymptotes on a log-log-scale is theoretically identical to the horizontal shift of the minimum (see Figure 3). Therefore, it is hypothesized that the equiluminant point can also be estimated from the sensitivities for the modulation ratios 1∶0 and 0∶1 (the asymptotes). In Figure 3 the measured sensitivity differences between the single blue modulation [log(contrast ratio) -∞] and single red modulation [log(contras ratio) ∞] are plotted as a function of the horizontal position of the minimum as obtained from the curve fits. Indeed, there is a strong correlation between these two values (r = 0.87, p<0.001 for central measurements; r = 0.76, p<0.001 for peripheral measurements; r = 0.84, p<0.001 for all measurements). The linear regression results in slopes of 0.87 and 0.93 for central and peripheral fixation respectively and a slope of 0.99 if the regression is performed on both sets of data simultaneously (see Figure 3); the slope is therefore close to the theoretically expected value of 1.

Although the individual photometric matches derived either from the modulation ratio for which sensitivity was minimal or from the differences in sensitivity at the asymptotes correlated well (r = 0.87 for central measurements and r = 0.76 for peripheral measurements, p<0.001 for both), the MPOD values calculated from the differences between the respective central and peripheral measurements did not reach statistical significance (r = 0.38, p = 0.069). Further, the MPOD estimates derived from the minima of the curves fitted to the sensitivity function correlated to a higher level of significance with MPR estimates than those derived from the asymptote differences (r = .59; p = 0.003 vs. r = 0.48; p = .018).

#### Calculation of MPOD from the HMP data

**Figure 4:**
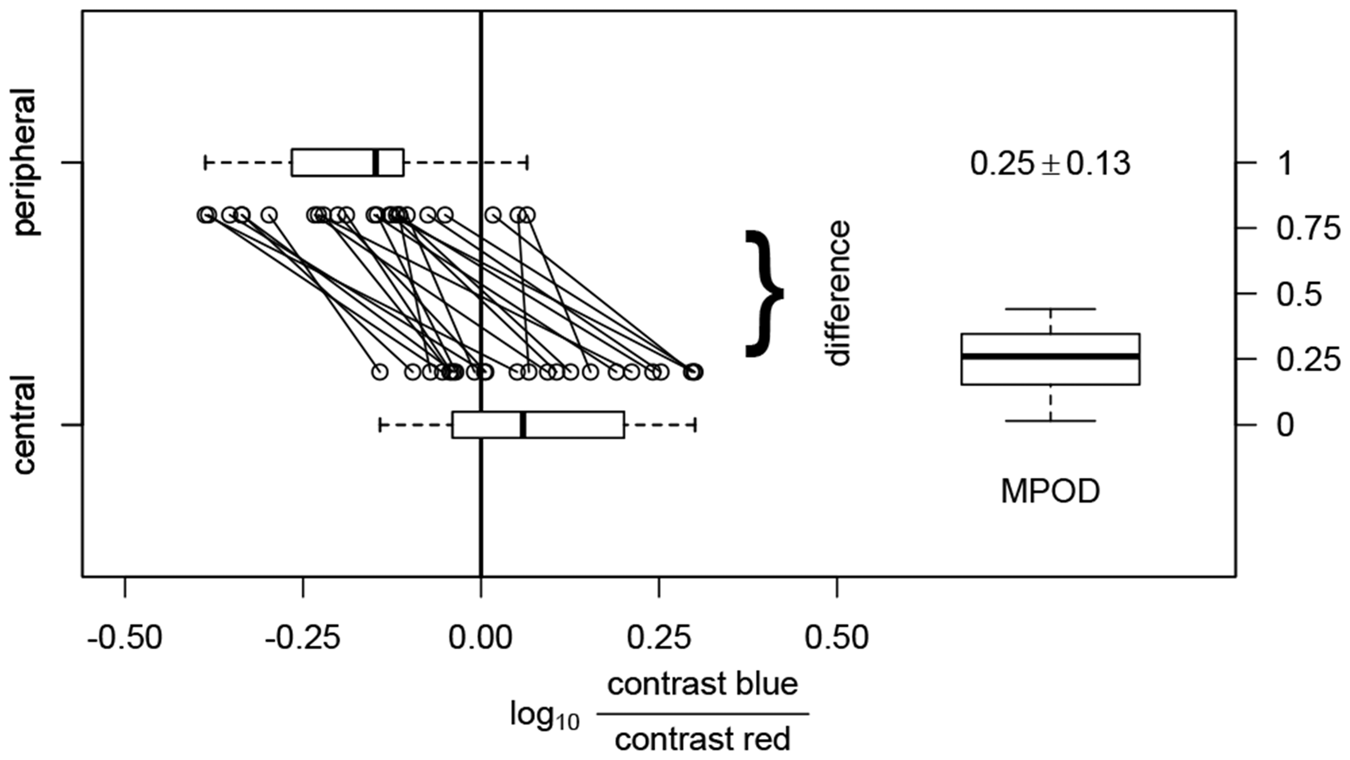
Photometric matches for central (bottom) and peripheral fixation (top) and the resultant MPOD values. Under the assumption that only macular pigment is responsible for differences in luminous sensitivity, the MPOD is calculated as the difference between the HMP minimum points.

Individual macular pigment optical densities (MPOD_HMP_) were estimated from the difference of the equiluminant settings for central and peripheral fixation. In Figure 4, these settings are shown for each of the 24 observers. Values from central and peripheral measurements were approximately normally distributed (Shapiro-test: p = 0.16 for central and p = 0.40 for peripheral measurements). Central equiluminance settings clustered around a zero contrast ratio, indicating a V_λ_-like spectral sensitivity although *the mean value differed significantly from zero (mean ± sd: 0.08±0.14, p = .01; student t-test).* The standard deviation is larger than observed for population measures of photometric matches between a middle and long wavelength lights, reflecting the added variation from interobserver MPOD differences in our measurements[21], [33]–[36]. Peripheral equiluminance settings were at significantly smaller contrast ratios than central measurements (mean ± sd: −0.17±0.13, p<.001 vs. central measurements; student t-test). This was the case for nearly all observers. The MPOD estimates were also normally distributed (Shapiro-test: p = 0.35) and had a mean of 0.25±0.13.

Thresholds for each contrast ratio were calculated as the mean of two threshold measurements (one starting at maximum, the other at zero contrast). To get an indication of measurement reliability, we calculated the MPOD separately only from the measurements starting at maximal contrast and from the ones starting at zero contrast for each subject. These two estimates correlated significantly with a correlation coefficient of 0.86 (p<0.001).

### Correlations with two established techniques

**Figure 5:**
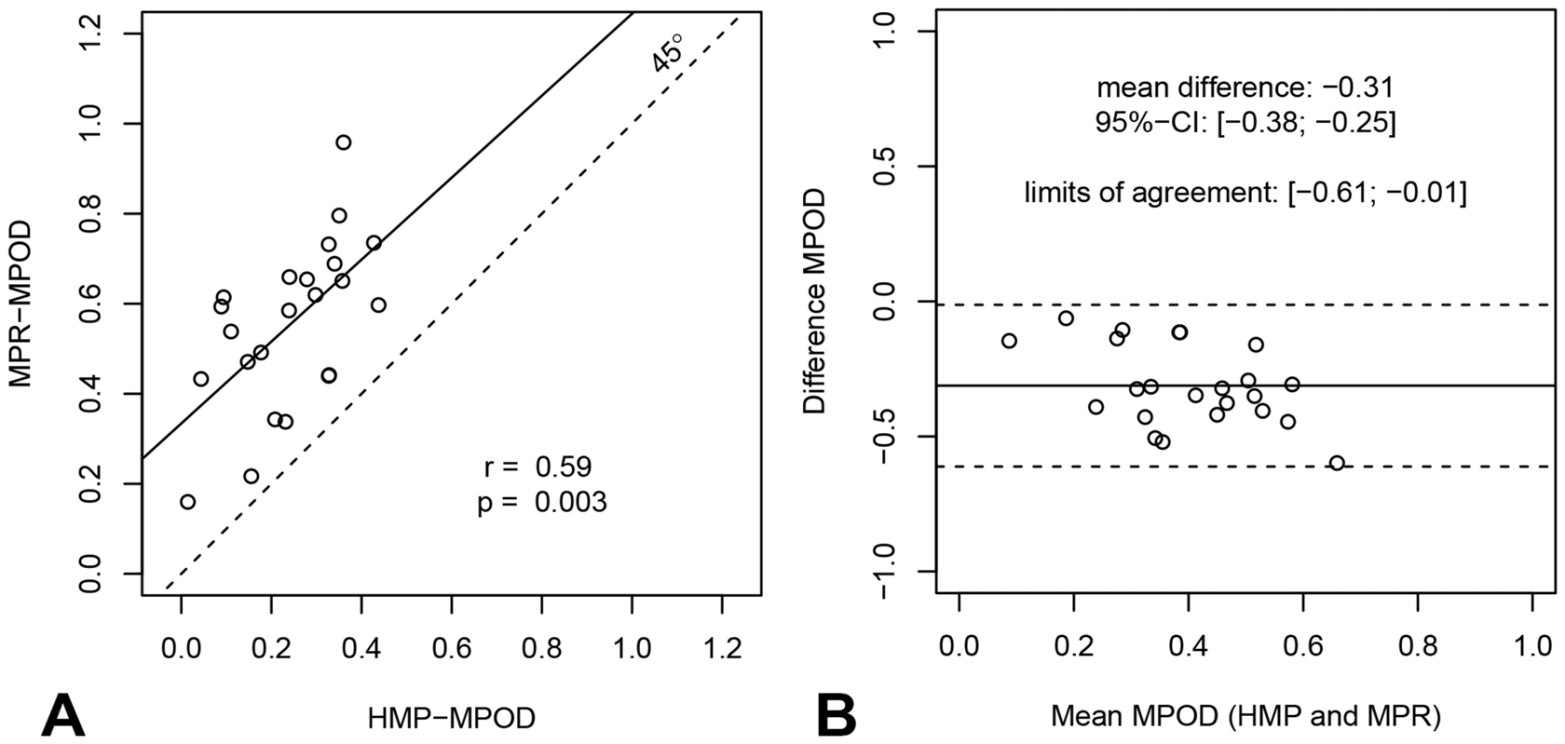
Agreement between MPOD measured with heterochromatic modulation photometry and with macular pigment reflectometry. a) The relationship between MPOD measured with HMP and with MPR. There is a significant positive correlation. b) The Bland-Altman plot shows that MPOD-HMP is systematically smaller than MPOD_MPR_: the mean difference is −0.31 with 95%-confidence interval of [−.38, −.25].

**Figure 6:**
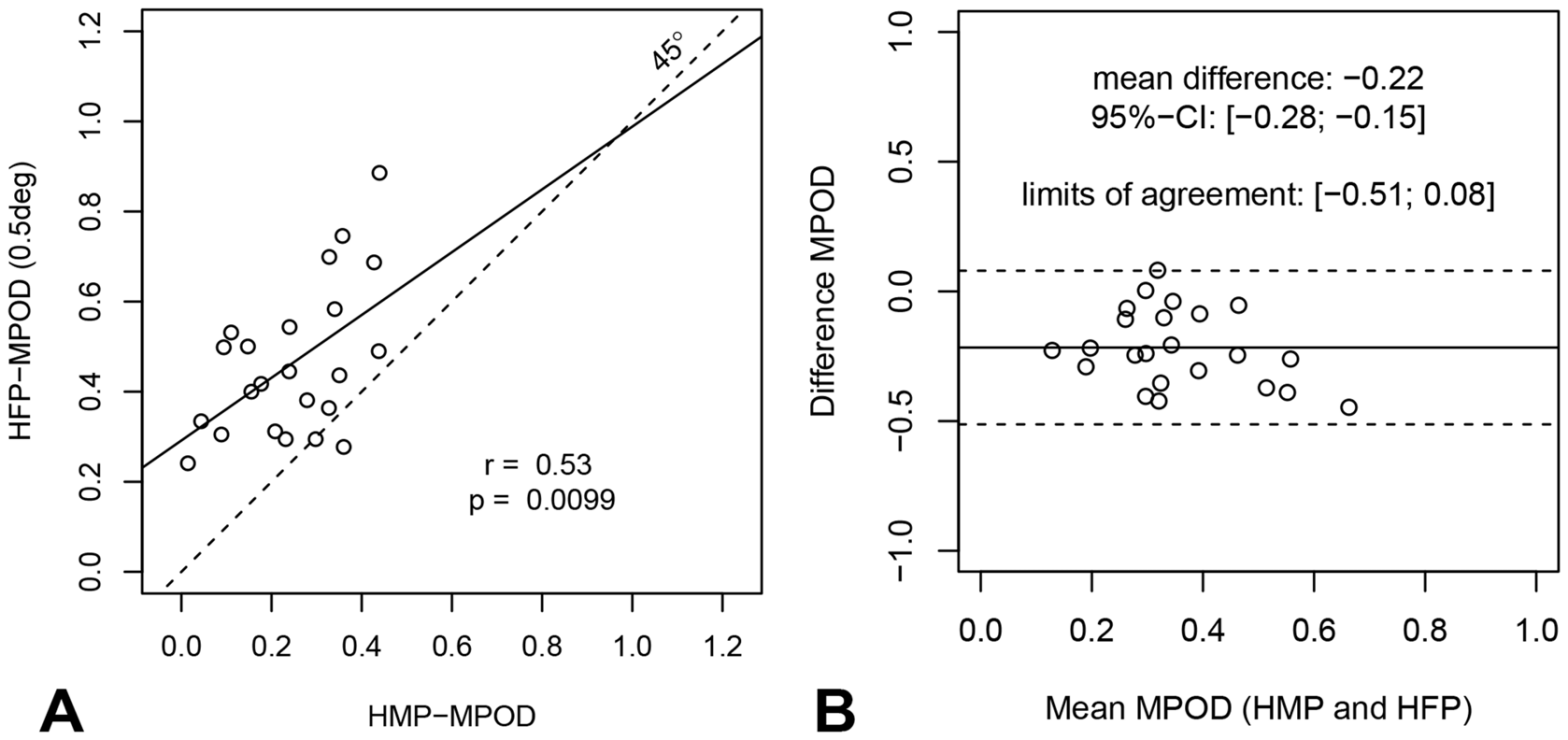
Agreement between MPOD measured with heterochromatic modulation photometry and with heterochromatic flicker photometry. a) The relationship between MPOD measured with HMP and with HFP (the latter measured with a 0.5° diameter ring). A significant correlation is found with HFP measurements at 0.25° and 0.5° retinal eccentricities, but not at 1.0° and 1.75°. b) The Bland-Altman plot shows that MPOD_HMP_ values were systematically smaller than MPOD_HFP_ at 0.5°: mean difference −0.22 with 95%-confidence interval of [−.28, −.15].

MPOD_HMP_ significantly correlated with MPOD_MPR_ (r = 0.59; p = .003, see Figure 5) and with MPOD_HMP_ measured at eccentricities of 0.25° (r = 0.54, p = .007) and 0.5°(r = 0.53, p = .010; see Figure 6), but not for measurements taken at 1.0° and 1.75°.

As can be seen in Table 1 and from the Bland-Altman plots (shown in Figure 5 and Figure 6), the MPOD estimates based on HMP were smaller than those based on either MPR or HFP. On average, MPOD_HMP_ was 0.31 OD smaller than MPOD_MPR_ (95%-confidence interval [−.38, −.25]) and 0.22 units smaller than MPOD_HFP_ at 0.5° eccentricity (95%-confidence [−.28, −.15]). As shown in the Bland-Altman plots, results are distributed evenly around the average difference and the differences are not larger at extreme mean MPOD values. It may be noted that the MPOD values obtained with the two published methods (MPR and HFP) are not significantly correlated with each other at any eccentricity.

**Table 1:** Macular pigment optical density (MPOD) values measured by different techniques.

Method		Mean ± SD	Shapiro-Test
Heterochromatic Modulation Photometry	MPOD	0.25±0.13	0.350
Macular Pigment Reflectometry	MPOD	0.56±0.18	0.769
	Lutein (L) optical density	0.21±0.07	<0.01
	Zeaxanthin (Z) optical density	0.35±0.14	0.017
	Zeaxanthin Ratio	0.61±0.15	<0.001
Heterochromatic Flicker Photometry	MPOD at 0.25°	0.57±0.18	0.452
	MPOD at 0.5°	0.47±0.16	0.125
	MPOD at 1.0°	0.36±0.14	0.375
	MPOD at 1.75°	0.19±0.10	0.803

## Discussion

Our data indicate that the optical density of macular pigment (MP) can be measured with HMP. We were generally able to achieve very good photometric matches. Although, as expected from the variability of macular pigment optical density reported in the literature [10], there was considerable individual variation in the position of the HMP minimum point, the average value for the observers tested was close to zero for central fixation and, as expected, were shifted to lower values of log [contrast blue/contrast red] for peripheral fixation for all observers. We expected the interindividual variability of the central measurements to be much higher than in the periphery, because only the former should be influenced by MPOD variability. When looking closely at the boxplots in Figure 4, one will find that the distance between the 25%- and 75%-quartile (that is left and right border of the box) is much smaller for the peripheral measurements, whereas the distance of the 5%- and 95%-quartiles (the “whiskers”) from the median is much larger. This raises the possibility that for a relevant number of subjects our expectations were met, but that a few subjects (∼8) displayed larger variability, possibly due to problems with eccentric fixation. On average, the sensitivities to short-wavelengths light in our study population were similar to that of a 2° standard observer [37], [38] in the central retina, whereas the sensitivities to short wavelengths were higher at the peripheral location.

The MPOD values measured with HMP correlated significantly with those from the two published techniques. Therefore, these findings suggest that our MPOD measurements using HMP can yield meaningful estimates. However, in our study, as in previous studies, the correlation between different methods was low and there were marked systematic differences between methods. On average, the HMP values were much lower than the MPR values and lower than the HFP values at 0.25°, 0.5° and 1°. This is expected, given to the larger diameter of the HMP stimulus. There were moderate correlations between HMP and either HFP or MPR, but the correlation between HFP and MPR was weaker and not statistically significant. The weak correlation between MPOD_HFP_ and MPOD_MPR_ is in line with a number of studies, reporting correlation coefficients of 0.42 [39], 0.56 [32] and 0.61 [40]. More recent studies that have used the modified HFP paradigm by Murray et al. have found better correlations with coefficients of 0.78 [16] and 0.72 [41].

It has been proposed [10] that MPOD measurements based on psychophysical methods are mainly determined by the edge of the stimulus, in contrast to reflectrometric methods where measurements integrate over the whole test field. The individual comparison of HMP and HFP data from our study does not support the ‘edge theory’ because the correlation between MPOD_HMP_ and MPOD_HFP_ was significant for the circular HFP stimuli with borders at 0.25° and 0.5° retinal eccentricity, but not for the annular HFP stimulus with the edge at 1° eccentricity (the HMP stimulus was circular with a 1° radius). Furthermore, MPOD_HMP_ correlated much better with MPR measured zeaxanthin optical density than with lutein optical density (see Table 2). Zeaxanthin is located predominantly in the center of the fovea [41]. This supports evidence, provided by Bone and coworkers [42], that questions the validity of the edge theory. On the other hand, more in line with the edge theory, the average magnitude of MPOD measured by HMP is lower compared with MPR that integrates over the whole central retina up to 0.5° eccentricity.

**Table 2:** Correlation between macular pigment optical densities measured by different methods.

	HMP	HFP at 0.25°	HFP at 0.5°	HFP at 1.0°	HFP at 1.75°
HMP	**–**	**0.54 (0.007)** *	**0.53 (0.010)** *	**0.42 (0.045)**	**not significant**
MPR-MPOD	**0.59 (0.003)**	not significant	not significant	not significant	not significant
MPR-LOD	0.47 (0.025)	not significant	not significant	not significant	not significant
MPR-ZOD	0.56 (0.006)	not significant	0.42 (0.040)	0.49 (0.017)	not significant

Significant correlations are reported with the Pearson R correlation coefficient and the p-values (without Bonferroni correction) in brackets. The correlations between HMP and MPR as well as HMP and HFP are the primary outcome measures (**bold**). MPOD measured with HMP correlates significantly with MPOD-MPR, and also with MPOD-HFP at two locations; however, only the correlation with HFP at 0.25° remains significant after Bonferroni correction for testing at four locations.

*The significances of the correlations with HFP have to be corrected for testing at four locations. The positive correlations with HFP at 0.25° and at 0.5° remain significant even after Bonferroni correction.

Differences between the HMP and HFP measurements can be explained only partly by the different stimulus conditions. The HFP technique uses annular stimuli for the measurements at 1° and 1.75°. Bone et al. found higher MPOD estimates for circular compared with annular stimuli of the same outer diameter [42]. Along this line, one would have expected higher MPOD estimates by HMP compared with HFP at 1°. This was not the case. However, reference measurements were performed less eccentrically in HMP than in HFP (6° vs. 7°). Therefore, residual macular pigment densities at 6° may have led to underestimation of MPOD in our HMP measurements.

To identify the exact location of the photometric matches (that is the point of minimum sensitivity) a model was fitted to the HMP data. The model is an elaboration of the original model, taking into account preretinal absorption [15], [17], [21]. The modified model accounts for the asymmetries in the heights of the asymptotes in the modulation-sensitivity-plots, which we found to be larger in observers with large shifts in the equiluminant point from the average of our observers. As outlined above, the new model predicts that the difference in height between the asymptotes equals the horizontal shift of the minimum on a log-log-scale. This is in line with our data (Figure 3). Therefore, MPOD estimation from HMP could theoretically be obtained from measurements of the asymptotes only, with precise measurements of modulation thresholds for two single conditions. This would simplify the procedure, shorten the testing time, and make the task easier to perform. However, the mathematical model is based on the assumptions that the psychophysical response depends linearly on modulation depth and is additive for the two stimulus wavelengths. It would be necessary to ensure that these requirements are met.

Our experimental conditions deviate from other HFP protocols for measuring MPOD by using a long-wavelength (660 nm) rather than the more typical middle-wavelength (540−570 nm) reference light. Although the long-wavelength reference increases the range of equiluminance settings, in gathering pilot data we found that observers reported a clearer flicker detection threshold, and the measured modulation-sensitivity-curves exhibited a more pronounced minima.

Other methods have been employed to overcome the limitations of HFP for MPOD measurement. To circumvent problems with broad or absent null-flicker zones, a so-called customized HFP was developed to provide an algorithm for determining the optimal frequency settings by measuring the CFF [19], [20], [43]. Inverse yoking of the luminances (i.e. balancing the luminance change of one light by a reciprocal luminance change in the other) allows the time-averaged luminance (but not chromaticity) to be kept constant[18]–[20]. Murray and coworkers [16] developed a paradigm for measuring MPOD that is in many aspects similar to HMP. Test lights are presented at a series of different luminance ratios, and flicker frequency is adjusted at each to estimate the critical flicker fusion frequencies (CFF). CFF is plotted against the luminance ratio and, similar to HMP, the horizontal position minimum of this curve indicates the relative sensitivity. Their paradigm also replaces the relatively complicated matching process in HFP by the simpler task of indicating the presence or absence of the appearance of flicker, and it generates a curve that enables the examiner to make judgments about the internal validity of the measurements. However, the mean chromaticity of the test field still changes during the experiment.

We do not claim that this change in chromaticity is always relevant. In fact, a comparison between in vitro and in vivo absorption spectra by Bone et al. [4], the very good agreement between HFP measurements and MPR measurements in the study by Van der Veen and coworkers [41] and a large number of clinical trials[8]–[10], [20], [43] supports the utility of HFP for measuring MPOD.

### Conclusion

MPOD can be estimated using HMP, and HMP has features that are superior to classic HFP protocols. Although, other modifications of HFP also offer some advantages, HMP is the only method where measurements are made at a constant mean chromaticity. The large number of studies that have successfully used HFP protocols to measure MPOD suggest that this advantage is not practically relevant under normal circumstances, but it cannot be ruled out that it may influence measurements for example in subjects with severe retinal disease. We believe HMP to be a useful method of measuring MPOD, both for basic studies of visual performance and in more clinically oriented investigations.

## Supporting Information

Table S1 **Raw data from the heterochromatic modulation photometry (HMP) measurements.**
(CSV)

Table S2 **Results from the macular reflectometry (MPR) and the heterochromatic flicker photometry (HFP) measurements, as well as clinical data.**
(CSV)

Table S3 **Variable names used in tables S1 and S2.**
(DOC)

Appendix S1 **Mathematical derivation of the augmented theoretical model for the relationship between modulation ratio and sensitivity.**
(PDF)
