# Supplementary material for: Macular Pigment Optical Density Measured by Heterochromatic Modulation Photometry
Source: PLoS One. 2014 Oct 29;9(10):e110521. doi: 10.1371/journal.pone.0110521 (PMC4212909; doi:10.1371/journal.pone.0110521)
Supplement: Table S3 — Variable names used in tables S1 and S2. (DOC) [file pone.0110521.s003.doc]

# Variable names used in the supplementary tables S1 and S2:

Table S1: Data HMP.

| Observer | Number of the observer |
| --- | --- |
| Fixation | central / peripheral |
| Frequency | Frequency in Hz |
| Threshold 1 | Threshold measured by the down staircase |
| Threshold 2 | Threshold measured by the up staircase |
| Mean Threshold | Mean of Threshold 1 and Threshold 2 |
| Contrast Red | Temporal contrast of the red LED |
| Contrast Blue | Temporal contrast of the blue LED |
| Sensitivity | Contrast Blue divided by Mean Threshold;  If Contrast Blue is zero, it is Contrast Red divided by Mean Threshold |
| X | Position on the X axis;  Usually log10(Contrast Blue / Contrast Red),  if either Contrast Blue or Contrast Red is zero, it is set to +/- 4 |
| Y | Position on the Y axis;  log10(Sensitivity) |

Table S2: MPOD values derived from HMP, MPR and HFP, as well as clinical and demographic data.

| Observer |  |
| --- | --- |
| Sex |  |
| Age |  |
| MPOD.HMP | MPOD estimate using HMP; difference between “Central” and “Peripheral” |
| MPOD.HMP.AS | MPOD estimate using only the HMP measurements at contrast ratios of 0:1 and 1:0 |
| Central | Central equiluminant point |
| Peripheral | Peripheral equiluminant point |
| MPOD.MPR | MPOD estimate using MPR; mean of several measurements (see N.MPR) |
| SD.MPOD.MPR | Standard deviation of MPOD estimates by MPR |
| Zeaxanthin | Zeaxanthin optical density measured by MPR |
| SD.Zeaxanthin |  |
| Lutein | Lutein optical density measured by MPR |
| SD.Lutein |  |
| ZRatio | Zeaxanthin Ratio |
| SD.ZRatio |  |
| N.MPR | Number of MPR measurements performed for this subject |
| MPOD.HFP.0.25 | MPOD estimate using HFP at 0.25 degree eccentricity; mean of several measurements |
| SD.0.25 |  |
| MPOD.HFP.0.5 |  |
| SD.0.5 |  |
| MPOD.HFP.1 |  |
| SD.1 |  |
| MPOD.HFP.1.75 |  |
| SD.1.75 |  |
| Sph.Equ | Observer’s refraction (spherical equivalent) |
| AQ.min | Results from the anomaloscope |
| AQ.max |  |
| RetinalThickness | Retinal Thickness determined by OCT at the thinnest part of the foveola |
