# Supplementary material for: Macular Pigment Optical Density Measured by Heterochromatic Modulation Photometry
Source: PLoS One. 2014 Oct 29;9(10):e110521. doi: 10.1371/journal.pone.0110521 (PMC4212909; doi:10.1371/journal.pone.0110521)
Supplement: Appendix S1 — Mathematical derivation of the augmented theoretical model for the relationship between modulation ratio and sensitivity. (PDF) [file pone.0110521.s004.pdf]

Appendix S1

Original Equations

The equations from the original paper are [1, 2]:

$$C = \left| \frac{(R_{max} + B_{min}) - (R_{min} + B_{max})}{2(R_{average} + B_{average})} \right| \tag{1}$$

$$A = \frac{C}{M_t} \tag{2}$$

where C is the contrast, $R_{min}/R_{max}/R_{average}$ are the minimum/maximum/average luminances of the red primary respectively, $B_{min}/B_{max}/B_{average}$ are the same for the blue primary, A is the psychophysical response amplitude, and M_t is the optimal modulation threshold for the subject.

Minor changes in Equation (1)

R_{max}, R_{min}, B_{max}, and B_{min} can be replaced by the average luminances ($R_{average}$, $B_{average}$) and the Michelson contrasts of modulation of the primaries (C_{red} and C_{blue}):

$$\begin{aligned} R_{max} &= R_{average} \cdot (1 + C_{red}) \\ R_{min} &= R_{average} \cdot (1 - C_{red}) \\ B_{max} &= B_{average} \cdot (1 + C_{blue}) \\ B_{min} &= B_{average} \cdot (1 - C_{blue}) \end{aligned} \tag{3}$$

Combining Equations (1) and (3) results in:

$$C = \left| \frac{R_{average} \cdot C_{red} - B_{average} \cdot C_{blue}}{R_{average} + B_{average}} \right| \tag{4}$$

Expressing $R_{average}$ as a multiple of $B_{average}$

In contrast to the original papers, we are considering $B_{average}$ and $R_{average}$ to be the subjective luminance of the observer that can deviate from the luminance of the standard observer because of large deviations in the cone fundamentals owing to different preretinal absorption. Therefore, when the primaries are set to identical CIE luminances, $B_{average}$ can be expressed as a multiple of $R_{average}$, where h corresponds to the change in subjective luminance:

$$R_{average} = 10^h \cdot B_{average} \tag{5}$$

However, the function is shape invariant only for vertical translations, but no longer for horizontal translations, i.e. changes in h. Combining Equations (4) and (5) results in:

$$C = \left| \frac{10^h \cdot C_{red} - C_{blue}}{10^h + 1} \right| \tag{6}$$

Changing equations (2) and (3), so that we get a sensitivity function y=f(x) on a log-log-scale

To plot the relationship as a function of the logarithm of the (C_{blue}/C_{red}) modulation ratios (x), one needs to find values for C_{red} and C_{blue} that can be entered into equation (3), so that:

$$\frac{C_{blue}}{C_{red}} = 10^x \tag{7}$$

We set the contrast of the LEDs so that:

$$C_{red} + C_{blue} = 1 \tag{8}$$

For example, if x=0.3 (this corresponds to a modulation ratio C_{blue}:C_{red} = 2:1) and if the sum of the modulation depths is 1, this results in the modulation depths $C_{blue} = 2/3$ and C_{red}=1/3.

From equations (7) and (8), it can be derived that

$$C_{red} = \frac{1}{1+10^x} \quad and \quad C_{blue} = \frac{10^x}{1+10^x} \tag{9}$$

The position on the y-axis corresponds to the psychophysical response amplitude and the flicker sensitivity

Now the psychophysical response amplitude can be expressed as (equations 2, 6 and 9):

$$A = \left| \frac{\left(\frac{10^h}{1+10^x}\right) - \left(\frac{10^x}{1+10^x}\right)}{10^h + 1} \right| / M_t = \left| \frac{10^h - 10^x}{(1+10^x)(1+10^h)} \right| / M_t \tag{10}$$

where M_t is the optimal modulation threshold for the subject.

Observe that A=0 when x=h and that $A = \frac{1}{1+10^h}$ when $x = \infty$ and that $A = \frac{10^h}{1+10^h}$ when $x = -\infty$. Thus, the ratio between the amplitudes at the asymptotes (for single red and single blue modulation) equals 10^h.

The function is plotted on a coordinate system with a logarithmic y-axis and the following variables are defined:

$$v = -\log M_t \quad and \quad y = \log A \tag{11}$$

where v is a vertical translation factor that depends on the subject's optimal modulation threshold. It follows that the function relating the logarithm of sensitivity to the logarithm of modulation ratio is (equations 10 and 11):

$$y = \log \left| \frac{\left(\frac{10^h}{1+10^x}\right) - \left(\frac{10^x}{1+10^x}\right)}{10^h + 1} \right| + v \tag{12}$$

Now y is expressed as a function of x with h and v corresponding to the horizontal and vertical scaling factors in the classic HMP publications.

Rectifying the sensitivity function

The equation has to be modified to account for rectifying of the function at the minimum sensitivity measured to account for residual flicker perception when the resultant luminance flicker is very small, possibly owing to intrusion from other mechanisms such as the frequency doubled response in the magnocellular pathway at equiluminance [3–5].

Final equation

The final equation is

$$y = \log \left| \frac{\left(\frac{10^h}{1+10^x}\right) - \left(\frac{10^x}{1+10^x}\right)}{10^h + 1} \right| + v \qquad with \;\; y = S_{min} \;\; for \;\; y < S_{min} \tag{13}$$

where S_{min} is the minimum sensitivity from the data set.

This function y=f(x) with the parameters h and v can be fitted to a set of data using a standard least-square algorithm for nonlinear functions.

References

1. Pokorny J, Smith VC, Lutze M (1989) Heterochromatic modulation photometry. JOSA A 6: 16181623.

2. Pokorny J, Jin Q, Smith VC (1993) Spectral-luminosity functions, scalar linearity, and chromatic adaptation. J Opt Soc Am A 10: 13041313.

3. Kremers J, Lee BB, Kaiser PK (1992) Sensitivity of macaque retinal ganglion cells and human observers to combined luminance and chromatic temporal modulation. J Opt Soc Am A 9: 14771485.

4. Schiller PH, Colby CL (1983) The responses of single cells in the lateral geniculate nucleus of the rhesus monkey to color and luminance contrast. Vision Res 23: 16311641.

5. Lee BB, Martin PR, Valberg A (1989) Nonlinear summation of M- and L-cone inputs to phasic retinal ganglion cells of the macaque. J Neurosci 9: 14331442.
